# Rheological Characterisation and Processability Window of Denim-Derived Cellulose Solutions in NMMO for Fibre Spinning

**DOI:** 10.3390/polym18091094

**Published:** 2026-04-30

**Authors:** Mostafa Akhlaghi Bagherjeri, Mehran Namjoufar, Abu Naser Md Ahsanul Haque, Milad Laghaei, Maryam Naebe

**Affiliations:** 1 Institute for Frontier Materials, Faculty of Science, Engineering and Built Environment, Deakin University, Geelong, VIC 3216, Australia; s222411544@deakin.edu.au (M.A.B.); s224254957@deakin.edu.au (M.N.); a.haque@deakin.edu.au (A.N.M.A.H.); 2 School of Science, RMIT University, Melbourne, VIC 3000, Australia; milad.laghaei@rmit.edu.au

**Keywords:** denim waste recycling, NMMO monohydrate, rheological characterisation, viscoelastic behaviour, shear-thinning

## Abstract

N-methylmorpholine N-oxide (NMMO monohydrate) is widely used for cellulose fibre production, as in the Lyocell process. However, fibre spinning from denim wastes remains significantly more complex due to its higher viscosity, the presence of indigo dye, and NMMO’s temperature sensitivity. These factors together create serious challenges for denim dissolution and fibre regeneration. This study presents a comprehensive rheological and structural characterisation of regenerated cellulose fibres derived from waste denim dissolved in NMMO. Oscillatory and steady-state rheological tests were conducted across concentrations (4–8 wt%) and temperatures (60–90 °C) to determine optimal spinning conditions. A 6% denim/NMMO solution at 80 °C displayed the most favourable rheological balance within the investigated concentration window (4–8 wt%), moderate complex viscosity, well-defined viscoelastic transitions, and a Tan δ value (~0.94) consistent with stable jet formation in air-gap spinning. Steady shear tests confirmed strong shear-thinning behaviour and mechanical predictability, essential for spinneret extrusion. Thermal ramp experiments validated 80 °C as the upper safe limit, balancing flow processability with structural integrity while avoiding solidification or NMMO degradation. The identified rheological parameters fall within ranges reported for spinnable cellulose dopes in air-gap spinning systems, suggesting strong potential for fibre formation under controlled conditions. These findings establish a robust rheological framework for denim-derived cellulose in NMMO and provide a foundation for future investigations into controlled fibre spinning and process scale-up in sustainable textile recycling.

## 1. Introduction

The increasing demand for sustainable alternatives in the textile industry has prompted a resurgence of interest in regenerated cellulosic fibres, particularly those derived from waste materials [1]. Among textile waste, denim, a durable cotton-based fabric, constitutes a significant portion due to its high global consumption and low recycling efficiency. The transformation of denim waste into high-performance regenerated fibres offers a viable solution to environmental and resource challenges, aligning with circular economy goals and sustainable manufacturing practices [2].

Cellulose cannot be melt-processed, and its dissolution is hindered by strong intra- and intermolecular hydrogen bonding, necessitating the use of specialised solvent systems [3,4,5,6]. Various solvent systems have been explored for cellulose dissolution, including N,N-dimethylacetamide/lithium chloride (DMAc/LiCl), ionic liquids (ILs), alkali-based systems, and N-methylmorpholine N-oxide (NMMO). Among these, NMMO is considered one of the most industrially viable due to its non-derivatising mechanism, recyclability, and established use in the Lyocell process [7,8,9,10,11,12]. Unlike the conventional viscose process, which involves toxic chemical intermediates and a high environmental burden [13,14,15,16], NMMO enables direct dissolution of cellulose without chemical modification. However, its application remains challenging due to high temperature requirements and sensitivity to thermal degradation [7,8,17,18,19].

These challenges are further amplified when processing post-consumer denim waste. The presence of indigo dye and residual textile finishing agents can interfere with cellulose–solvent interactions, affect dissolution efficiency, and significantly alter the rheological behaviour of the resulting solutions. As a result, achieving consistent processability and spinnability in denim-derived cellulose/NMMO systems requires careful control of solution properties and processing conditions.

A key factor in fibre spinning is the rheological behaviour of the cellulose dope. Parameters such as complex viscosity (|η*|), storage modulus (G′), and loss modulus (G″) govern flow behaviour, viscoelastic balance, and jet stability during spinning [20,21,22,23,24]. In particular, the interplay between viscous and elastic responses determines the ability of the solution to undergo deformation while maintaining filament integrity. These rheological properties are highly sensitive to temperature, concentration, and processing history, all of which influence chain mobility, entanglement density, and structural stability.

Temperature plays a critical role in controlling the processability of cellulose/NMMO solutions. Increasing temperature generally reduces viscosity and improves flow. However, excessive thermal exposure may lead to cellulose degradation or solvent instability [17]. Conversely, lower temperatures can result in premature solidification due to NMMO crystallisation, limiting processability. Therefore, identifying an optimal temperature–concentration window is essential for maintaining a balance between flowability and structural integrity.

Fibre formation from cellulose solutions typically relies on dry-jet wet spinning, where both viscosity and viscoelasticity must be carefully controlled to ensure stable jet formation and filament drawability [25,26,27]. In NMMO-based systems, temperature control is particularly critical, as rapid cooling can lead to premature solidification and process instability. The use of an air gap between the spinneret and coagulation bath enables thermal stabilisation of the extruded filament prior to regeneration, providing improved control over fibre formation conditions.

Despite significant progress in cellulose dissolution and fibre regeneration, most studies focus either on spinning outcomes or on rheological characterisation of well-defined cellulose sources such as wood pulp. Limited attention has been given to establishing rheology–processability relationships for post-consumer textile waste, particularly indigo-dyed denim, where residual dyes and finishing agents can alter solution behaviour. Furthermore, discrepancies often arise between rheologically optimised conditions and practical spinning trials, especially when key parameters such as temperature and residence time are not adequately controlled. This highlights the need for a systematic rheology-driven framework to guide process design prior to fibre spinning.

Therefore, this study aims to evaluate the temperature- and concentration-dependent rheological behaviour of denim-derived cellulose/NMMO solutions and to define a rheological processability window relevant to fibre spinning. Direct fibre spinning under fully controlled and rheologically optimised conditions is beyond the scope of the present study and will be addressed in future work. By systematically analysing viscoelastic and flow properties, this work seeks to provide a predictive framework for processing denim waste into regenerated cellulose fibres. The influence of indigo dye on cellulose dissolution and rheological behaviour has been investigated in our previous work [2], where direct comparisons between denim-derived cellulose and pure cellulose were performed under identical conditions. It was demonstrated that the presence of indigo reduces the diffusion coefficients of system components and leads to higher solution viscosity compared to pure cellulose systems, as supported by both experimental rheology and molecular dynamics simulations. Therefore, indigo is expected to influence the rheological behaviour of denim/NMMO solutions, although such effects are not independently evaluated in the present study.

## 2. Materials and Methods

### 2.1. Materials

For this study, NMMO monohydrate (98% purity) and propyl gallate (PPG, 98%) were sourced from Merck Life Science Pty Ltd., Bayswater, VIC, Australia. Additionally, discarded blue denim made from 100% cotton fibre [2,28] was utilised as the cellulose source.

### 2.2. Preparation of Denim Powder

Waste denim used in this study was previously characterised for degree of polymerisation (16,500 ± 313) and molecular weight (2673 ± 51 kg/mol), which was supported by parallel molecular dynamics simulation [2]. The denim used contains residual indigo dye, the influence of which on cellulose dissolution and solution behaviour has been previously characterised through comparative studies with pure cellulose systems [2]. A significantly higher degree of polymerisation than control cellulose (372 ± 10) was observed, which was attributed to the presence of indigo, indicating that it consists of more complex and larger molecules than pure cellulose [1,2,28].

In this study, denim was processed into snippets through a rotary cutter mill (Pulverisette 19, Fritsch GmbH, Idar-Oberstein, Germany) using a 0.2 mm sieve. Subsequently, these snippets were further ground in an Attritor mill (S/1, Union Process, Akron, OH, USA) for 6 h to produce a denim slurry. To obtain fine denim powder, the slurry was dried using a B-290 mini spray dryer (Buchi Labor-technik AG, Flawil, Switzerland) with a feed rate of 10 mL/min. The inlet and outlet air temperatures during spray drying were maintained at 180 °C and 100 °C, respectively [2,28]. It should be noted that the mechanical process was designed to reduce particle size and improve dissolution behaviour, rather than to chemically purify the cellulose, and the powder may contain residual impurities such as sizing agents and finishing chemicals. The chemical and structural characteristics of the resulting denim powder, including the presence of residual indigo and the preservation of cellulose structure, have been comprehensively characterised in our previous work [2]. Fourier transform infrared spectroscopy (FTIR) confirmed the preservation of characteristic cellulose functional groups, with observed shifts in O–H stretching indicating changes in hydrogen bonding and crystallinity. X-ray diffraction (XRD) analysis showed a reduction in crystallinity and a transition from cellulose I to cellulose II after processing. Furthermore, ^13^C NMR spectroscopy revealed disruption of interchain hydrogen bonding within the cellulose structure following dissolution and regeneration. Importantly, residual indigo dye was retained within the system as an inherent component of the denim-derived cellulose.

Therefore, the material used in this study should be considered as a realistic representation of recycled denim-derived cellulose rather than a purified cellulose system, and the reported rheological behaviour reflects this composition.

### 2.3. Denim/NMMO Solution Preparation

In each experiment, a specific amount of denim powder was dispersed in 20 g of NMMO (containing 50% water) in a 150 mL flask. To prevent oxidation of the NMMO during heating, PPG was added at a concentration of 0.06% *w*/*w* at the start of the process, simultaneously with the denim powder at the different concentrations (4, 6, and 8 *w*/*w*%). The mixture was then stirred at 390 rpm using a mechanical stirrer equipped with a hotplate (LabCo Scientific, Brendale, QLD, Australia) and heated in an oil bath at 50 °C for 4 h to allow the fibres to swell. Following this pre-treatment phase, the temperature was raised to 70 °C and maintained for 45 min to ensure complete dissolution of the cellulose. The selected concentration range (4–8 wt%) was chosen based on preliminary dissolution feasibility and the viscosity limits of the rheometer geometry at elevated temperatures. Concentrations below 4 wt% produced insufficient chain entanglement for meaningful viscoelastic analysis, while concentrations above 8 wt% exhibited limited processability under the applied dissolution conditions. Therefore, the present study focuses on identifying a practical rheological processability window rather than establishing a fully optimised concentration interval.

### 2.4. Characterisation

Rheological measurements were performed using a TA Instruments DHR-3 rotational rheometer (TA Instruments, New Castle, DE, USA) equipped with a parallel-plate geometry (25 mm diameter, smooth plates). The plate gap was set to 50 μm, and measurements were conducted under gap-controlled mode. Prior to testing, freshly prepared denim/NMMO solutions were carefully loaded onto the lower plate at the target test temperature, excess material was trimmed, and a solvent trap was used to minimise moisture loss and solvent evaporation during high-temperature measurements. Following sample loading, the solution was allowed to equilibrate for 5 min at each measurement temperature (60, 70, 80, and 90 °C) to ensure thermal and structural stabilisation. All rheological tests were conducted under a nitrogen atmosphere to minimise oxidative degradation of NMMO. Oscillatory frequency sweep tests were performed at a constant strain amplitude within the linear viscoelastic region (LVR), which was determined by preliminary strain sweep measurements. Because complex viscosity (|η*|) is frequency dependent for viscoelastic polymer solutions [29], the representative values reported in this study were extracted from the low-frequency region (0.1–1 rad s^−1^), where the response most closely reflects zero-shear behaviour relevant to fibre spinning conditions. Frequency sweeps were conducted over an angular frequency range of 0.1–100 rad s^−1^, and the G′, G″, |η*|, and Tan δ were recorded as functions of frequency and temperature. Steady shear flow measurements were carried out at 80 °C using a controlled shear rate mode over a shear rate range of 0.01–100 s^−1^ to evaluate non-Newtonian flow behaviour and shear-thinning characteristics relevant to fibre spinning. Temperature ramp experiments were conducted by increasing the temperature from 60 °C to 140 °C at a controlled heating rate, while monitoring the evolution of complex viscosity. Each rheological experiment was performed in duplicate, and representative data are reported. Data acquisition and analysis were carried out using TA TRIOS 5.7.2 software. Crossover frequencies (G′ = G″), zero-shear viscosity, and Tan δ values were extracted directly from the processed datasets without additional smoothing unless otherwise stated.

## 3. Results

### 3.1. Experimental Observations

**Figure 1:**
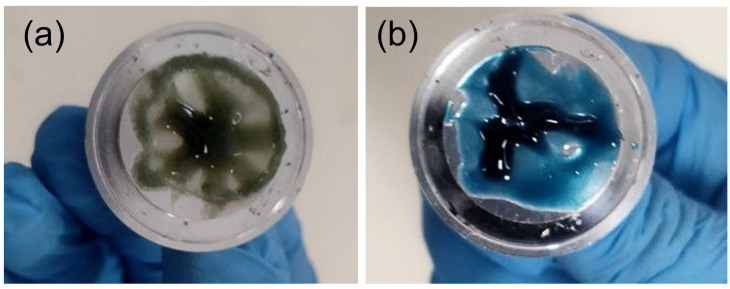
Visual comparison of denim/NMMO solution before and after implementing the improved stabilisation strategy. Solution prepared with late-stage PPG addition and high-temperature exposure (105 °C) shows discolouration from blue to green, indicating oxidative degradation of NMMO and potential cellulose breakdown (**a**). Solution prepared with early-stage PPG incorporation (0.06 wt.%) and moderate thermal treatment (max 70 °C) remains visually clear and stable, confirming effective suppression of thermal and oxidative degradation during prolonged processing. The blue colour of the solution is due to the indigo dye present in the denim waste (**b**).

In our previous study, denim-based films were fabricated by directly casting freshly prepared solutions onto glass substrates using a film applicator, followed by immediate immersion in water for 12 h to regenerate the cellulose structure. In that procedure, no prolonged thermal exposure or post-preparation stirring was involved. The denim/NMMO solution was prepared by dissolving blue denim powder in NMMO at 50 °C for 5 h, followed by a temperature increase to 105 °C, at which point 0.02 wt.% PPG was introduced as an antioxidant to mitigate the oxidative degradation of NMMO at elevated temperatures. The initial blue colour observed in the denim–NMMO solution is primarily due to the presence of indigo dye in the denim waste. However, for rheological characterisation and air-gap spinning, the solutions must remain thermally stable and processable for extended periods (typically 2–3 h) after preparation. Under these conditions, we observed a gradual colour transition in the solution, shifting from blue to light green and eventually to a brownish shade, indicating the onset of NMMO oxidation and potential thermal degradation of cellulose. (Figure 1a). This degradation compromises the molecular integrity of the solution, potentially altering its rheological profile and diminishing fibre-forming performance during spinning. To overcome this limitation, we developed a modified preparation strategy aimed at enhancing the oxidative stability of the denim/NMMO solution. In this method, both the denim powder and 0.06 wt.% PPG, an antioxidant stabiliser, was added to the NMMO solvent at the beginning of the solution preparation process. The mixture was maintained at 50 °C for 4 h to allow for gradual swelling and initial dissolution of the cellulosic material. Subsequently, the temperature was increased to 70 °C and maintained for 45 min to complete the dissolution process and obtain a homogeneous, clear, and processable solution (Figure 1b).

Unlike earlier approaches, where PPG was added only after heating to higher temperatures (105 °C) and the solution was processed immediately after preparation, the current method ensures that antioxidant protection is present throughout the entire preparation phase, including the critical early stages when oxidative species may begin to form. Importantly, this strategy also avoids excessive temperatures, which are known to accelerate NMMO degradation and cellulose depolymerisation, both of which can be visually detected through colour changes (e.g., green to brown) and negatively impact rheological properties.

Following the completion of denim/NMMO solution preparation, the sample was kept under continuous stirring and heating at 70 °C for the duration of the rheological testing, which typically lasted approximately three hours. This holding period reflects the time frame required for practical rheological assessments and mimics the expected processing time during air-gap spinning. The solution remained visually stable and free from discolouration, indicating successful inhibition of oxidative degradation.

This revised protocol not only ensures the chemical stability of the denim–NMMO system but also maintains consistent rheological performance necessary for reliable measurement and successful fibre formation. The effectiveness of early-stage antioxidant incorporation combined with moderate thermal conditions highlights a practical and scalable route for processing regenerated cellulose solutions from denim-derived cellulose, particularly under conditions that require extended thermal exposure.

### 3.2. Oscillatory Rheology Interpretation of Denim/NMMO Solutions

**Figure 2:**
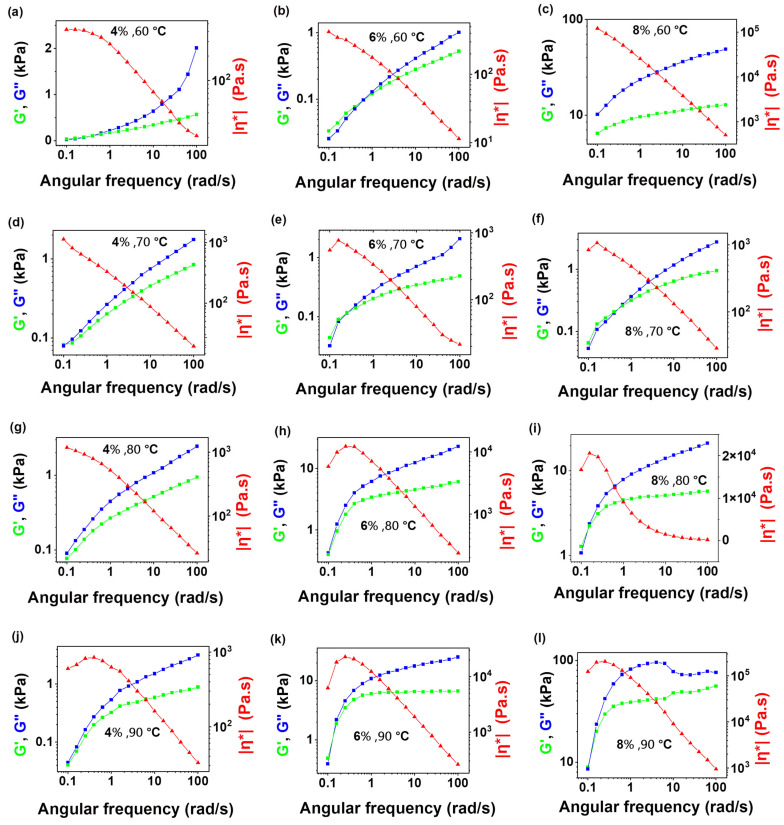
Frequency sweep results for denim–NMMO solutions at different concentrations and temperatures: (**a**–**c**) 4 wt%, 6 wt% and 8 wt% denim at 60 °C; (**d**–**f**) 4 wt%, 6 wt% and 8 wt% denim at 70 °C; (**g**–**i**) 4 wt%, 6 wt% and 8 wt% denim at 80 °C; (**j**–**l**) 4 wt%, 6 wt% and 8 wt% denim at 90 °C. Different coloured lines in graphs showing respective coloured Y-axis information.

Oscillatory rheological measurements were employed to evaluate the viscoelastic behaviour of denim-derived cellulose solutions in NMMO at concentrations of 4 wt%, 6 wt%, and 8 wt% across a temperature range of 60 °C to 90 °C. As shown in Figure 2, the G′, G″, and |η*| displayed frequency-dependent trends typical of entangled polymer systems, where G″ exceeded G′ at lower frequencies, indicative of a viscous-dominated response while G′ surpassed G″ at higher frequencies, signifying a transition to more elastic-like behaviour. Such frequency-dependent crossover behaviour has been widely reported for concentrated denim/NMMO solutions and is generally attributed to the formation of transient entanglement networks arising from intermolecular hydrogen bonding and chain overlap [30,31].

A systematic shift in the crossover point (G′ = G″) toward lower angular frequencies was observed with increasing cellulose concentration and decreasing temperature. This trend is consistent with classical polymer solution theory, where increased concentration enhances entanglement density and prolongs relaxation times, thereby shifting viscoelastic transitions to lower frequencies. Similar behaviour has been documented for both NMMO-based Lyocell dopes and ionic liquid cellulose systems processed via dry-jet wet spinning [25,32].

The 8 wt% denim/NMMO solutions exhibited crossovers at 0.1–0.5 rad/s, with complex viscosities exceeding 10^4^–10^5^ Pa·s, consistent with a highly structured network suitable for producing strong fibres but potentially challenging in terms of flow through the spinneret. In contrast, 4 wt% solutions exhibited crossovers near 0.7–1.0 rad/s and much lower viscosities (500–2000 Pa·s), which improved flowability but compromised jet stability during spinning. The 6 wt% solutions achieved the most favourable rheological profile among the concentrations investigated, particularly at 80 °C, exhibiting crossover around 0.5–0.7 rad/s and a representative complex viscosity of approximately 8000 Pa·s measured in the low-frequency region (0.1–1 rad s^−1^), where |η*| approaches a quasi-plateau behaviour characteristic of entangled cellulose/NMMO systems suitable for spinning evaluation. This rheological window falls within the range reported for stable dry-jet wet spinning of regenerated cellulose fibres, where |η*| values of 10^3^–10^4^ Pa·s and crossover frequencies below 1 rad s^−1^ are commonly associated with balanced spinnability [25,26]. The selection of the 60 °C to 90 °C temperature window was informed by the thermal characteristics of the NMMO–cellulose system. At lower temperatures (<60 °C), there is an increased risk of solution solidification due to the crystallisation of NMMO monohydrate, impeding homogeneous mixing and extrusion. Conversely, at temperatures above 90 °C, the risk of thermal degradation becomes significant as documented in mechanistic studies of the Lyocell process [17]. Therefore, careful thermal control is essential to preserve molecular integrity while maintaining adequate flowability.

These findings, supported by Figure 2, highlight the importance of precise tuning of concentration and temperature to achieve optimal viscoelastic behaviour. When benchmarked against established cellulosic solutions in the literature [25], the rheological characteristics of the 6 wt% denim/NMMO solution at 80 °C fall within the ideal range (|η*| ≈ 10^3^–10^4^ Pa·s; crossover < 1 rad/s) for effective fibre spinning via the air-gap method. This validates the potential of repurposing indigo-dyed denim waste into high-performance regenerated cellulose fibres, provided the rheological window is carefully defined and maintained.

### 3.3. Evaluating Viscoelastic Damping Profiles of Denim/NMMO Solutions

**Figure 3:**
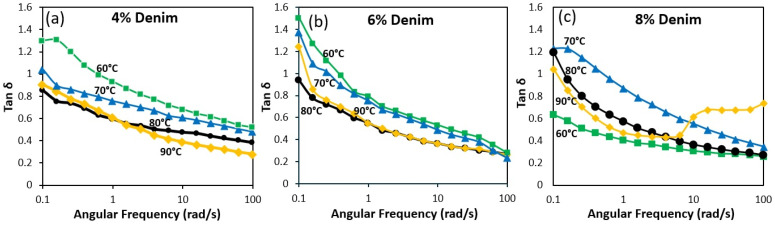
Tan δ (G″/G′) as a function of angular frequency for 4 wt% (**a**), 6 wt% (**b**), and 8 wt% (**c**) denim/NMMO solutions at different temperatures (60–90 °C).

The viscoelastic damping behaviour of denim/NMMO solution was further examined using Tan δ (G″/G′) as a function of angular frequency, providing insights into the energy dissipation and storage characteristics of the solutions under oscillatory shear. As shown in Figure 3, Tan δ curves were obtained for 4 wt% (Figure 3a), 6% (Figure 3b), and 8 wt% (Figure 3c) denim solutions at 60 °C, 70 °C, 80 °C, and 90 °C. Across all concentrations, a consistent pattern was observed: Tan δ values were higher at low frequencies, indicating viscous-dominated behaviour, and decreased monotonically with increasing frequency, reflecting a shift toward more elastic response under rapid deformation. This frequency-dependent transition is characteristic of polymer solutions with entangled networks and is crucial for predicting spinnability during fibre formation. Similar frequency-dependent damping profiles have been reported for cellulose dissolved in NMMO and ionic liquids, where entanglement-controlled relaxation governs viscoelastic transitions [26,31,32]. The 8% denim/NMMO solutions (Figure 3c) demonstrated the most structured viscoelastic behaviour up to 80 °C, with Tan δ values decreasing significantly from low to high frequencies. The Tan δ peak at 80 °C dropped to approximately 0.63, indicating a strong elastic contribution to the viscoelastic response, which has been widely associated with improved filament stability during air-gap spinning of cellulose solutions, where balanced viscous and elastic behaviour is required to sustain extensional deformation without jet breakage [29,31]. However, at 90 °C, the Tan δ curve deviated from this trend, showing a non-monotonic behaviour where values decreased initially but then rebounded at higher frequencies. This anomaly signals a breakdown in the elastic network, likely due to thermal degradation or disruption of cellulose–solvent interactions [17], and marks 90 °C as a critical threshold where the solution loses structural integrity.

Similarly, the 6 wt% denim/NMMO solutions (Figure 3b) exhibited ideal damping behaviour at 80 °C, with Tan δ peaking at approximately 0.94. This profile signifies a well-balanced viscoelastic state where flowability is preserved without compromising elasticity, making it the most favourable condition among the investigated formulations [25,32]. At lower temperatures (60 °C and 70 °C), the Tan δ values remained above 1.0, suggesting insufficient elastic recoil for stable fibre formation. At 90 °C, a moderate increase in Tan δ to around 1.15 was observed, indicating the early onset of structural weakening.

In contrast, 4 wt% denim/NMMO solutions (Figure 3a) maintained comparatively higher Tan δ values at low frequencies indicating limited entanglement density. Although Tan δ decreased at higher frequencies, the magnitude of elastic dominance remained lower than that observed for 6 wt% and 8 wt% systems. Such behaviour is consistent with dilute-to-semi-dilute transition regimes in cellulose/NMMO solutions, where insufficient chain overlap limits network formation [30]. Thus, although the 4% solution shows improvement at higher frequencies, its rheological profile remains less favourable for stable fibre spinning compared to 6 wt% and 8 wt% formulations.

The Tan δ results, therefore, confirm that 80 °C is the optimal processing temperature for denim/NMMO solution, particularly at 6 wt% concentration. Under this condition, the viscoelastic profile achieves the best balance between energy dissipation and elastic recovery, supporting conditions typically associated with stable jet elongation and fibre formation during air-gap spinning. Although no universal critical Tan δ value exists for fibre spinning systems, previous studies on regenerated cellulose solutions have shown that solutions exhibiting Tan δ values close to unity generally provide a favourable balance between viscous flow and elastic recovery required for stable jet elongation during dry-jet wet spinning.

### 3.4. Steady Shear Rheology of 6 wt% Denim/NMMO Solution: Yielding, Non-Newtonian Flow, and Shear-Thinning Dynamics for Fibre Spinning

Since 80 °C and 6 wt% were identified as the optimal processing conditions based on previous viscoelastic analyses, all subsequent rheological characterisations were conducted using the 6 wt% denim/NMMO solution at this temperature. This focus ensures that the findings are directly relevant to the most spin-friendly formulation identified for fibre production via air-gap spinning. The applied shear-rate window (0.01–100 s^−1^) was selected to encompass the range typically encountered during spinneret extrusion in dry-jet wet spinning systems reported in the literature. In particular, shear rates between approximately 1 and 50 s^−1^ are commonly associated with industrial cellulose/NMMO spinning processes and therefore provide a relevant basis for evaluating processability under realistic extrusion conditions.

**Figure 4:**
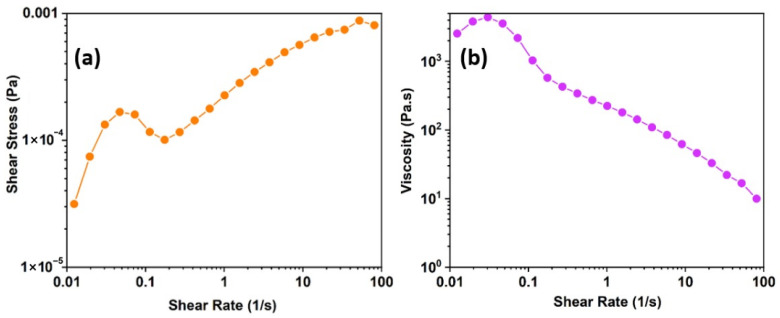
Steady shear rheological behaviour of 6% denim/NMMO solution at 80 °C: shear stress versus shear rate curve (**a**), and viscosity as a function of shear rate (**b**).

The steady-state rheological profile of the 6 wt% denim/NMMO solution demonstrates complex, yet favourable, non-Newtonian behaviour characteristic of entangled polymer systems designed for fibre spinning [30,31]. The shear stress versus shear rate curve (Figure 4a) reveals a distinctive yield-like response at low shear rates (0.01–0.1 s^−1^), where stress initially increases sharply, reaches a local maximum, and then dips slightly before continuing to rise. This non-monotonic shape is a hallmark of transient structural rearrangements, often referred to as “stress overshoot,” commonly observed in viscoelastic biopolymer solutions. It reflects the temporary disruption and recovery of physical entanglements and weak microstructural associations within the cellulose-rich network [17].

At higher shear rates (>0.2 s^−1^), the stress increases smoothly with shear rate, indicating stable flow without catastrophic structural breakdown. The sub-linear increase observed in the log–log representation confirms shear-thinning behaviour. Shear-thinning in cellulose solutions arises from progressive alignment of polymer chains in the flow direction and reduction in intermolecular friction, a phenomenon widely documented in Lyocell-type spinning solutions [31,32]. This behaviour is advantageous for fibre spinning, as it allows high resistance to deformation at rest while reducing viscosity under extrusion conditions. The viscosity profile (Figure 4b) further supports this interpretation. The solution exhibits a steady-shear viscosity of approximately 3000 Pa·s at low shear rates (<0.1 s^−1^). Although a well-defined zero-shear viscosity plateau was not observed within the experimentally accessible shear-rate range, this low-shear viscosity level remains consistent with values reported for entangled denim/NMMO solutions capable of maintaining filament coherence prior to spinneret extrusion in air-gap spinning systems, consistent with a well-entangled network capable of maintaining filament coherence in the air-gap region. As the shear rate increases from 0.01 to 100 s^−1^, viscosity decreases by nearly two orders of magnitude. Similar magnitudes of shear-thinning have been reported for spinnable cellulose/NMMO systems and are considered essential for achieving manageable extrusion pressures while preserving sufficient elastic contribution for jet stability [25]. Importantly, no abrupt viscosity drops or discontinuities were observed across the measured shear range, suggesting that the network structure undergoes progressive alignment rather than catastrophic breakdown. This behaviour is consistent with the rheological requirements of dry-jet wet spinning, where controlled flow adaptation under shear must coexist with adequate structural integrity upon exiting the spinneret [32].

Overall, the 6 wt% denim/NMMO solution at 80 °C exhibits non-Newtonian characteristics comparable to those reported for industrially relevant Lyocell dopes. While the present observations are specific to denim-derived cellulose and direct comparison must consider differences in molar mass distribution and solvent composition, the combination of moderate zero-shear viscosity, pronounced shear-thinning behaviour, and absence of flow instabilities suggests that the system falls within the rheological regime compatible with fibre spinning applications.

### 3.5. Temperature-Dependent Complex Viscosity of 6% Denim/NMMO Solution: Defining the Optimal Processing Window for Air-Gap Spinning

**Figure 5:**
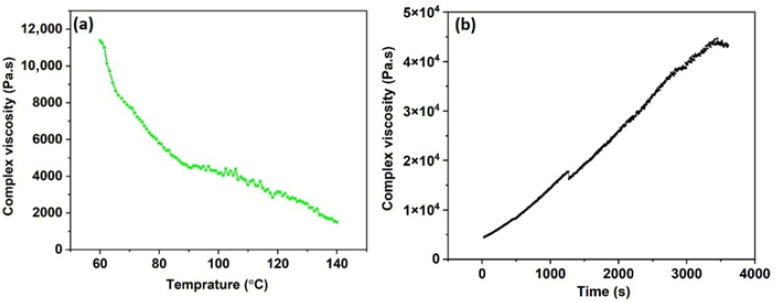
Temperature-dependent complex viscosity profile of 6% denim/NMMO solution measured from 60 °C to 140 °C (**a**), and time-dependent complex viscosity at 80 °C (**b**).

Following the identification of 6 wt% denim/NMMO at 80 °C as the optimal condition based on oscillatory rheology and Tan δ analysis, further temperature sweep testing was performed to understand the thermal response of the solution across a broader range. Figure 5a presents the evolution of |η*| as a function of temperature, revealing the characteristic thermal softening behaviour of entangled cellulose solutions.

At lower temperatures (60–70 °C), the solution exhibits a high complex viscosity of approximately 10^4^ Pa·s, indicative of a densely entangled molecular network. Similar temperature-dependent viscosity behaviour has been reported for Lyocell-type dopes, where increasing temperature enhances segmental mobility and reduces effective entanglement lifetime [31]. Elevated viscosity at these temperatures supports filament cohesion but may require higher extrusion pressures during spinning. As the temperature increases from 70 °C to 90 °C, |η*| shows a marked decrease to around 6000 Pa·s, demonstrating improved chain mobility and enhanced flow processability without fully compromising structural integrity, a range consistent with industrial benchmarks for Lyocell and other regenerated cellulosic dopes [33]. Industrial Lyocell processing is typically conducted within a comparable temperature window to balance viscosity reduction and structural stability [8].

Beyond 90 °C, the viscosity continues to decline, reaching a value around 2000 Pa·s by 140 °C. While lower viscosity facilitates flow through spinneret orifices, excessive thermal exposure can accelerate NMMO decomposition and cellulose depolymerisation, as documented in mechanistic studies of the Lyocell process [17]. Such degradation may compromise molecular weight distribution and, consequently, fibre mechanical properties.

Therefore, the optimal processing window is confirmed to lie between 70 and 90 °C, with 80 °C as the most favourable temperature. This region ensures a balance between sufficient entanglement to maintain fibre integrity and adequate flowability for spinneret extrusion. The observed trend in Figure 5a reinforces conclusions drawn from Tan δ profiles and crossover frequency shifts, validating 80 °C as the rheologically and thermally ideal condition for spinning denim-derived regenerated cellulose fibres using NMMO.

### 3.6. Time-Dependent Viscosity Evolution of 6% Denim/NMMO Solution: Evidence of Structural Maturation for Spinning Readiness

Figure 5b presents the time-dependent complex viscosity profile of the optimised 6 wt% denim/NMMO solution at 80 °C, providing insight into the time-dependent rheological evolution of the solution during thermal conditioning. An initial steady increase in viscosity is observed over the first 1200 s, reflecting enhanced molecular entanglement and chain interaction as the system equilibrates under heat. This phase likely corresponds to the alignment and swelling of cellulose chains, contributing to gradual network development.

Beyond the initial equilibration period, a gradual increase in complex viscosity is observed throughout the measurement window. Such time-dependent viscosity evolution has previously been reported for concentrated cellulose/NMMO systems and is commonly associated with progressive chain rearrangement and solvent redistribution under thermal conditioning [30,31]. However, it should also be noted that part of the observed viscosity increase may reflect structural recovery following sample loading and trimming in the rheometer geometry, which is typical for highly entangled polymer solutions. The absence of a clearly defined plateau within the experimental time window suggests that the system had not yet reached a fully steady rheological state. Therefore, the observed viscosity evolution is interpreted as a progressive structuring process rather than a fully stabilised equilibrium condition relevant to spinning readiness [32]. Nevertheless, the absence of abrupt viscosity drops or irregular fluctuations indicates that no significant thermal degradation or catastrophic network breakdown occurred during the measurement period at 80 °C.

In contrast, progressive viscosity increase may reflect redistribution of solvent within the polymer matrix and strengthening of intermolecular associations. However, it should be noted that prolonged holding at elevated temperature in NMMO systems can eventually promote degradation reactions if stabilisation is insufficient [17]. Therefore, controlled residence time is essential to balance structural maturation with chemical stability. However, it should be noted that the present study does not include direct thermal analysis to distinguish between physical structuring and potential chemical effects. Complementary techniques such as differential scanning calorimetry (DSC) or thermogravimetric analysis (TGA) could provide further insight into thermal transitions and stability, and this will be considered in future work.

From a processing perspective, time-dependent structuring can be advantageous in dry-jet wet spinning, where sufficient viscosity is required to maintain filament coherence in the air gap while still permitting extrusion through the spinneret. The observed viscosity evolution suggests that a controlled thermal holding step may contribute to progressive rheological stabilisation prior to spinning, although longer equilibration times would be required to confirm the establishment of a steady-state viscosity plateau. Nevertheless, further studies incorporating extensional rheology and industrial residence time simulation would be required to fully correlate time-dependent viscosity evolution with fibre mechanical performance.

### 3.7. Comparative Evaluation of Rheological Parameters in NMMO-Based Dope Systems

Contextualising the rheological performance of the denim/NMMO solution developed in this study, Table 1 presents a comparative summary of the relevant literature focused on regenerated cellulose dopes used for air-gap spinning. It should be noted that the comparison between different solvent systems is qualitative, as variations in cellulose source, molecular weight, and dissolution conditions across studies limit direct comparability. Therefore, the reported trends should be interpreted as indicative rather than strictly equivalent benchmarks. Although several solvent systems have been proposed, including various ionic liquids such as 1,5-diazabicyclo [4.3.0]non-5-enium acetate ([DBNH][OAc]) and 1-Ethyl-3-methylimidazolium acetate ([EMIM][OAc]), the benchmark for industrially viable systems continues to be centred around NMMO monohydrate due to its established use in Lyocell fibre production and its ability to generate high-tenacity fibres without introducing toxic co-solvents.

In this work, the 6 wt% denim/NMMO solution achieved a time-dependent zero-shear viscosity between ~8000 and 45,000 Pa·s, with crossover frequencies ranging from 0.5 to 1.0 rad/s and a G′ = G″ modulus of ~1000–3000 Pa. These values suggest a highly entangled polymer system with excellent elasticity and flow resistance, both critical parameters for dry-jet wet spinning. When compared to previous studies, these rheological properties closely align with those reported by Hermanutz et al. [27], who found a viscosity of 9914 Pa·s for cotton linters dissolved in NMMO at 12 wt% concentration. Despite the lower cellulose content in the current denim-based system, the resulting viscosity and elastic modulus are comparable or even superior, which may be associated with the presence of indigo residues. Our previous study [2] reported that the presence of indigo was associated with increased viscosity compared to pure cellulose systems. Molecular dynamics simulations suggested that indigo molecules may form additional interactions, which may contribute to reduced molecular mobility and increased resistance to flow. Experimentally, this effect was evident as denim solutions containing indigo reached solubility limits at lower concentrations (~10%) compared to pure cellulose systems, which remained soluble up to 18%. These findings explain the relatively high viscosity and elasticity observed in the current 6 wt% denim/NMMO solution despite its lower cellulose content.

Interestingly, Hauru et al. [26] also reported relatively low viscosities (~705 Pa·s) for 13% eucalyptus pulp in NMMO, with crossover frequencies significantly higher (1.5–8.3 rad/s), indicating a weaker elastic network than observed in the present study. This contrast demonstrates the unique behaviour of denim-derived cellulose in NMMO, which may benefit from residual textile treatments or differences in molecular weight distribution. Notably, this study’s most favourable concentration within the investigated range (6 wt%) and temperature (80 °C) produced viscoelastic properties superior to many higher-concentration systems, highlighting the effectiveness of pre-treatment and dissolution protocols tailored to textile waste.

In comparison to ionic liquid systems such as those used by Haslinger et al. [34] and Michud et al. [25], the denim/NMMO solution offers significantly higher viscosity at comparable or even lower polymer loadings. While IL systems demonstrate tunable viscosity and reduced processing volatility, they often require co-solvent systems or thermal stabilisers, which can complicate recycling and scalability. The higher processing temperatures and costs associated with ILs also present a barrier to large-scale adoption, especially for circular economy models targeting textile waste valorisation.

Moreover, it is worth emphasising that only a few studies have examined the rheology of NMMO solutions derived from non-virgin sources like denim. The data presented here thus contribute novel insights into the processing feasibility of recycled denim. The successful achievement of crossover viscosity in the ideal window (10^3^–10^4^ Pa·s), combined with strong elastic response, validates the denim/NMMO solution as a viable alternative to virgin pulp systems. Furthermore, the demonstrated rheological stability across a range of temperatures and time intervals suggests its robustness in industrial settings.

Overall, this study shows that denim waste, when dissolved in NMMO under optimised conditions, not only matches the rheological profiles reported for virgin cellulose but, in some respects, exceeds them. The findings advocate for broader consideration of post-consumer textiles as feedstock in fibre regeneration processes, supporting both environmental and economic sustainability goals. These insights position the denim/NMMO formulation as a strong candidate for integration into closed-loop fibre production pipelines.

## 4. Conclusions

This study investigates the rheological behaviour and processability of post-consumer denim-derived cellulose dissolved in NMMO monohydrate. The results demonstrate that solution behaviour is strongly influenced by cellulose concentration and processing temperature within the tested range. Among the formulations examined, the 6 wt% denim/NMMO solution at 80 °C exhibited the most favourable rheological balance within the investigated parameter space.

Analysis of Tan δ and crossover frequency indicated structured viscoelastic behaviour consistent with entangled cellulose systems reported in the literature. Steady shear and temperature sweep measurements further showed pronounced shear-thinning characteristics and thermally sensitive viscosity reduction typical of regenerated cellulose dopes. The temperature-dependent data suggest that processing above 90 °C may introduce instability risks associated with reduced viscosity and potential solvent–polymer degradation, although detailed chemical analysis was not performed in this study.

The identified rheological window provides a predictive framework for fibre spinning of denim-derived cellulose in NMMO. This is particularly for air-gap spinning systems where both viscosity and viscoelastic balance are critical. However, direct fibre spinning under fully controlled and rheologically optimised conditions was not performed in this study, and therefore, a direct correlation between rheological properties and fibre performance remains to be established.

It should be noted that a direct rheological comparison between denim-derived cellulose and pure cellulose was not performed in this study. However, such comparisons have been previously reported, demonstrating the influence of indigo on solution behaviour. Future studies incorporating intermediate concentrations (e.g., 5 wt% and 7 wt%) would further refine the definition of the spinnability window. Future work should also focus on translating the identified rheological conditions into controlled spinning trials incorporating temperature-regulated spinnerets, defined air-gap environments, and extensional flow analysis. Additionally, mechanical characterisation of regenerated fibres and evaluation of process scalability will be necessary to validate the applicability of denim-derived cellulose as a sustainable feedstock for regenerated fibre production.

Overall, this study establishes a robust rheological foundation for understanding and optimising denim/NMMO systems and provides a critical step toward the development of scalable, circular textile-to-textile recycling processes.

## Figures and Tables

**Table 1:** Comparative rheological properties of regenerated cellulose dopes in various solvent systems used for air-gap spinning.

Study	Solvent System	Cellulose Source	Conc. (%)	Zero-Shear Viscosity (Pa·s)	Crossover Frequency (rad/s)	G′ = G″ Modulus (Pa)	Spinning Method
6 wt% denim/NMMO (This work)	NMMO (monohydrate)	Recycled indigo-dyed denim	6%	8000 → 45,000 (time-dependent)	0.5–1.0	1000–3000	Target: Air Gap
[34]	[DBNH][OAc] (IL)	Cotton/polyester blends	6.5–10.5%	686–13,294	1.4–6.9	939–3224	Air Gap
[35]	[DBNH][OAc] (IL)	Eucalyptus kraft pulp	13%	11,551, 70 °C	2.31	5315	Air Gap
[26]	NMMO (monohydrate)	Eucalyptus pulp	13%	705	1.5–8.3	3708	Air Gap
[27]	NMMO (monohydrate)	Cotton linters	12%	9914	Not specified	Not specified	Air Gap + Wet
[33]	[DBNH][OAc] (IL)	Cotton/spruce blends	13–15%	25,565–38,034	0.8–1.5	3000–6000	Air Gap

## Data Availability

The original contributions presented in this study are included in the article. Further inquiries can be directed to the corresponding author.
